# Evaluation of the Real-World Effectiveness of Vaccines against COVID-19 at a Local Level: Protocol for a Test-Negative Case–Control Study

**DOI:** 10.3390/vaccines10050822

**Published:** 2022-05-23

**Authors:** Cátia Brazete, Marta Pinto, Lígia Sá, Ana Aguiar, Filipe Alves, Raquel Duarte

**Affiliations:** 1 EPIUnit—Instituto de Saúde Pública, Universidade do Porto, 4050-600 Porto, Portugal; ana.aguiar@ispup.up.pt (A.A.); raquel.duarte@arsnorte.min-saude.pt (R.D.); 2 Unidade de Saúde Pública do Alto Minho, 4904-459 Viana do Castelo, Portugal; ligia.sa@ulsam.min-saude.pt; 3 Unidade de Investigação Clínica da ARS Norte, 4000-477 Porto, Portugal; marta.pinto@arsnorte.min-saude.pt (M.P.); filipe.alves@arsnorte.min-saude.pt (F.A.); 4 Faculdade de Psicologia e Ciências da Educação, Universidade do Porto, 4200-135 Porto, Portugal; 5 Laboratório Para a Investigação Integrativa e Translacional em Saúde Populacional (ITR), 4050-600 Porto, Portugal; 6 Instituto de Ciências Biomédicas Abel Salazar, Universidade do Porto, 4050-303 Porto, Portugal; 7 Serviço de Pneumologia, Centro Hospitalar de Vila Nova de Gaia/Espinho, 4434-502 Vila Nova de Gaia, Portugal

**Keywords:** COVID-19 vaccines, effectiveness, case–control studies, district hospitals

## Abstract

Vaccines against COVID-19 approved for use in the EU/EEA have been shown to be highly effective against wild-type SARS-CoV-2. However, their effectiveness against new variants may be reduced. This study aims to evaluate the effectiveness of vaccines against COVID-19 in the prevention of symptomatic and severe disease, during pre- and post-omicron phases. Individuals who sought treatment at the emergency department of a Portuguese hospital with COVID-19-like disease and were tested for SARS-CoV-2 are the subjects of the study. Patients who received a positive result are considered cases, while those with negative results are the controls. The test-negative case–control method is one of the study designs recommended by WHO to estimate the effectiveness of vaccines against COVID-19. The main advantage of this design is that it controls for the healthcare seeking bias, commonly present in traditional cohort and case–control designs. This study may have broad implications for understanding the real-world performance of the COVID-19 vaccines at the local level, which may play a key role in promoting adherence to vaccination. Moreover, this study may contribute to inform decisions regarding booster doses and variant-specific vaccine formulations leading to the control of this and future pandemics.

## 1. Introduction

Vaccines against COVID-19 were launched as a promising tool in prevention and control of the current pandemic. As of 1 April 2022, there were 349 vaccines under development worldwide, of which 153 are in clinical trials [1]. The European Commission has already granted conditional marketing authorization for five vaccines, including RNA vaccines (i.e., Comirnaty and Spikevax), adenovirus vector vaccines (i.e., Vaxzevria and COVID-19 vaccine Janssen) and a recombinant protein vaccine (i.e., Nuvaxovid).

Vaccine performance can be assessed by efficacy, effectiveness and impact. A vaccine’s efficacy is its ability to reduce the infection or disease risk among the vaccinated in controlled and ideal conditions, usually estimated in randomized controlled trials [2]. Effectiveness is the reduction in infection or disease risk among vaccinated through vaccination in real-world conditions assessed in observational studies [2]. A vaccine’s impact comprises the reduction in the incidence of infection or disease in a population in which some of its members are vaccinated. The impact of a vaccine depends on the vaccination coverage and results: on the one hand of direct effects of vaccination in vaccinated people and, on the other hand, of any indirect effect on vaccinated and non-vaccinated people through herd immunity [3,4]. The impact may also relate to other variables beyond the disease, such as health systems’ functioning and socioeconomic indicators.

According to the World Health Organization (WHO) guidelines, the primary efficacy point estimate should be at least 50% so that a vaccine is approved [5]. COVID-19 vaccines already authorized for use in European Union and European Economic Area achieved the efficacy of up to 96.4% in initial clinical trials [6,7,8,9,10]. After being considered efficacious in trials, experimental studies raise ethical concerns, as they need a control group that would be deprived from the benefits of vaccination [11]. Therefore, and according to the WHO’s recommendations, the performance of vaccines must also be evaluated under real-world conditions and using data from observational studies [2].

Two systematic reviews with meta-analysis showed that the vaccine effectiveness against the SARS-CoV-2 infection ranged from 70 to 90% for the wild-type strains [12,13]. However, concerns have been raised regarding reduced vaccine effectiveness against new variants of concern (VoC) [14].

**Figure 1:**
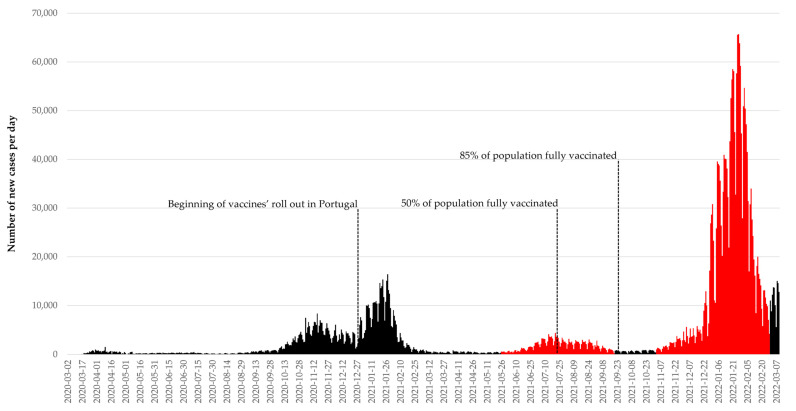
COVID-19 epidemic curve representing the evolution of the pandemic in Portugal. The periods for comparative analysis of COVID-19 vaccines effectiveness in this study are highlighted in red and correspond to the 4th and 5th pandemic waves.

As of 10 March 2022, Portugal had over three million cumulative laboratory-confirmed cases of SARS-CoV-2, emerging from five waves of infection, as can be seen in Figure 1 [15]. Delta variant (B.1.617.2) appeared in India in December 2020 and rapidly spread worldwide, becoming dominant by August 2021 in Portugal. It has been shown to be more transmissible and virulent than the Alpha variant [16]. Additionally, a study performed in Portugal showed significantly higher odds of vaccine breakthrough infection in Delta-infected patients than in Alpha-infected, suggesting lower effectiveness of the mRNA vaccines in preventing infection with the Delta variant [17], which is consistent with other studies [18,19].

The B.1.1.529 (Omicron) variant was first found in South Africa in November 2021, and it quickly spread worldwide. Omicron variant was shown to be 2.8-times more infectious than the Delta [20] and was responsible for the fifth pandemic wave in Portugal, which reached its peak on the 25 January 2022 with 5598 cases per million inhabitants. Omicron has shown immunity escaping capacity in laboratory studies and booster vaccine doses may be essential to overcome this [21,22].

This article presents the study protocol for evaluating the effectiveness of vaccines against COVID-19 in the prevention of symptomatic and severe disease in a district located in the northern region of Portugal, using a retrospective test-negative case–control design.

We will perform a comparative analysis between the fourth pandemic wave (1 June to 30 September 2021), dominated by Alpha and Delta variants and the fifth pandemic wave (1 November 2021 to 2 March 2022), Omicron-dominant, represented in Figure 1. In the beginning of the first period, the Alpha variant was dominant in Portugal, and by the week of 21 to 27 June 2021, the Delta variant became dominant [23]. The fifth wave of infection in Portugal, corresponding to our second period in analysis, was dominated by the Omicron variant since the week of 20 to 26 December 2021 [24].

The vaccination campaign in Portugal started on 27 December 2020. At that time, it only began with Comirnaty from Pfizer-BioNTech. In the first weeks of January, the first batch of Spikevax (previously COVID-19 vaccine Moderna) arrived in Portugal and soon began to be administrated. On the 7 February 2021, the first batch of Vaxzevria (previously COVID-19 Vaccine AstraZeneca) arrived, and on the 14 April 2021, the first batch of Jcovden (previously COVID-19 vaccine Janssen) was delivered in Portugal. Therefore, the four vaccines used in Portugal at that time were already in circulation in both periods of the study.

Monitoring mass-vaccination campaigns is essential for the prevention and control of emerging diseases. As new variants of concern emerge, early evidence on the effectiveness of vaccines is essential for informing policy decisions. Are booster doses effective? How long does their effect last? How should we prioritize booster doses over other non-pharmacological measures? Moreover, vaccine performances may be different when they are used in specific populations, different geographic settings and throughout different pandemic phases [2]. Thus, vaccines’ effectiveness should be assessed either at the local, regional, national and international levels.

## 2. Materials and Methods

We will perform an observational study using a retrospective test-negative case–control design. This method has been used successfully to estimate the effectiveness of vaccines against respiratory diseases such as influenza virus infection [25,26,27] and COVID-19 [28,29,30,31,32].

A test-negative study is considered a variant of the traditional case–control design: the study population is composed by individuals who have a predefined set of symptoms, seek help at the same healthcare unit and are tested by the same methodology, in this case, real-time reverse transcription polymerase chain reaction (rRT-PCR) or an antigen test for SARS-CoV-2. The cases are individuals who receive a positive result and controls are those with a negative result [2,33].

**Figure 2:**
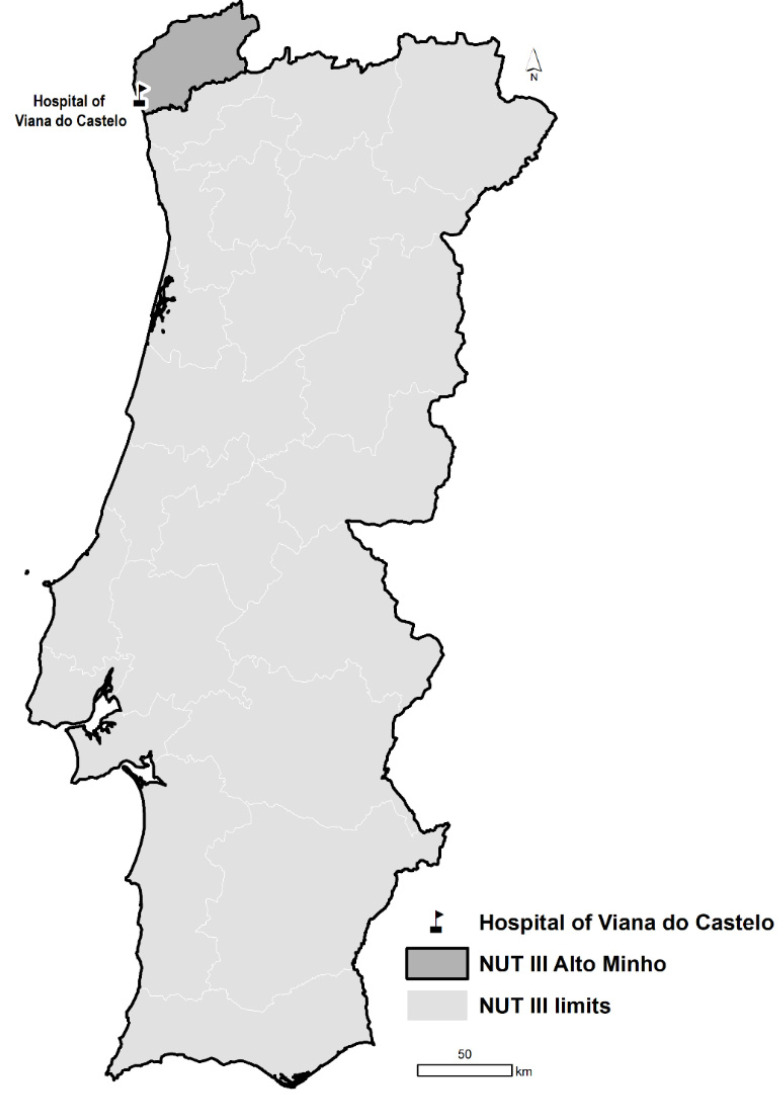
Location of Hospital of Viana do Castelo, in the Alto Minho NUTSIII region, on the northwest coast of Portugal.

Patients with COVID-19-like illness admitted to the Hospital of Viana do Castelo and Hospital of Ponte de Lima from 1 June 2021 until 2 March 2022, will be enrolled. These hospitals serve a population of 231,293 people resident in the district of Viana do Castelo, located in the northern region of Portugal, as shown in Figure 2.

Patients will be eligible if they are aged 18 years old or above on the date of hospital admission, resident in the district of Viana do Castelo, meeting any of the WHO clinical criteria for COVID-19, namely acute onset of fever, cough, general weakness, fatigue, headache, myalgias, sore throat, coryza, dyspnoea, recent onset of anosmia or dysgeusia, anorexia, nausea, vomiting, diarrhoea or altered mental status who received rRT-PCR or antigen testing for SARS-CoV-2 within 10 days of illness onset.

Exclusion criteria include patients not eligible for vaccination against COVID-19 or without information on the vaccination status and without SARS-CoV-2 test results available.

Primary outcomes include (1) SARS-CoV-2 symptomatic infection, confirmed by rRT-PCR or antigen test in respiratory samples from the nasopharynx or oropharynx and (2) moderate–severe COVID-19 disease, defined by hospitalization for over 24h, intensive-care unit (ICU) admission or death.

Baseline demographic and health data are going to be collected, namely age, gender, municipality of residence, date of vaccination and type of vaccine used, number of doses, previous SARS-CoV-2 infection, and being in a clinically extremely vulnerable group due to immunodepression or respiratory disease [34]. These variables are going to be studied as potential confounders, as they may influence both the receipt of the vaccine and the occurrence of COVID-19.

The results will be compared in two periods: 1 June to 31 September 2021, corresponding to the fourth pandemic wave in Portugal and 1 November 2021 until the 2 March 2022 (fifth pandemic wave). Vaccine’s roll-out was fast in Portugal, being 50% of people fully vaccinated by 20 July 2021. Moreover, on the 22 September 2021, Portugal was the first country in the world hitting the milestone of 85% of the population fully vaccinated [35]. Initially, COVID-19 vaccines were only approved for people older than 16 (Comirnaty) or 18 years old (the other vaccines approved in the UE/EEA). In Portugal, vaccination of adolescents between 12 and 15 years old begun in August and for children from 5 to 11 years old was only approved in December of 2021. Therefore, children and adolescents under 18 years will be excluded from this study.

Data analysis will be performed using R Statistical Package^®^, Vienna, Austria (SPSS), 4.1.0 version. We will perform a descriptive analysis of all variables obtained within the scope of this research project. For continuous variables with a symmetric distribution, the mean and standard deviation are going to be used; if the distribution is not symmetric, the median and interquartile range will be chosen. Categorical variables will be described as absolute and relative frequencies.

The normal distribution of the data will be verified by visual inspection of the histogram and the Kolmogorov–Smirnov test. Baseline characteristics of cases and controls will be compared using the chi-square test, Fisher’s test, Student’s *t* test or Mann–Whitney U test, depending on the nature of the variable. The association between the vaccination status and baseline characteristics will be investigated for cases and controls. The analysis will be stratified according to age, gender, municipality of residence, date of onset of illness, date of vaccination, vaccine administered, previous SARS-CoV-2 infection, and criteria for belonging to extremely vulnerable groups [34]. The presence of interaction will be evaluated by comparing the odds ratio (OR) in the various strata for a given variable. The presence of confounding will be assessed by comparing the unadjusted and adjusted OR for each of the covariates.

Multivariate logistic regression models will be used to estimate the unadjusted and adjusted OR for each of the covariates described, considering possible confounders. Covariates were selected a priori based on their known associations with SARS-CoV-2 infection or severe disease and inoculation with a COVID-19 vaccine [33] and will be evaluated as potential confounders.

The vaccine effectiveness (EV) will be estimated from the vaccination odds ratio, adjusted for confounders (OR_ad_), between cases and controls, according to the following formula:

OR = (1 − OR_ad_) × 100

The variables will be tested for multicollinearity. The presence of interaction will be verified through the likelihood test or Wald test and will be included in the model if they are significant for a significance level of 5%.

In a subsequent analysis, the vaccine effectiveness will be evaluated by the time since vaccination. The time since vaccination will be calculated by subtracting the test date from the last inoculation date. The time since vaccination will be modelled as a continuous variable.

The protocol of this study was approved by the Ethics Committee of the Local Health Unit of Alto Minho (ULSAM) with the reference number 05/2022. The ethical principles of human medical research contained in the Declaration of Helsinki, as well as national legislation, will be respected. The data collected will be aggregated and anonymized, guaranteeing the necessary confidentiality of the information collected. In addition, the principal investigator and her supervisors are subjected to medical confidentiality provided for in the Code of Ethics of the Portuguese Medical Association.

## 3. Results

On the 14 May 2022, the data collection was still ongoing, and it is expected to be completed by June 2022. A preliminary analysis is going to be performed after finishing data collection. Currently, we have obtained data on 1329 eligible patients (652 cases and 677 controls) for both periods of the study and we plan to increase the number by 30%. We expect to obtain a vaccine effectiveness of between 40 and 70% in the prevention of symptomatic infection and 70 and 90% in the prevention of severe cases of COVID-19, according to previously described results. It is anticipated that this effectiveness may be lower in more advanced age groups due to a less-robust response of the immune system in these individuals. Additionally, we expect lower vaccine effectiveness in unvaccinated or partially vaccinated people, particularly in people with moderate-to-severe COVID-19. As the waning of VE has been previously reported [36], we believe that VE will be lower in people vaccinated with their last dose first. However, data analysis will allow us to confirm this. The results will be disseminated in national and international scientific journals in 2022–2023.

## 4. Discussion

This project aims to evaluate the effectiveness of vaccination against COVID-19, to produce scientific evidence that can support strategies for the prevention and control of pandemics. This might contribute to the planning of more suitable vaccination campaigns and to tackle phenomena such as vaccine hesitancy [37].

This will be the first study to evaluate the real-world effectiveness of the vaccination campaign against COVID-19 in people over 18 years old in Portugal. The study design described has important advantages, namely: (1) the cases and controls are selected in the same health care unit and, therefore, probably residing in the same geographic area and in the same sociocultural environment, reducing bias due to risk variation according to the location [2]; (2) cases and controls all sought care for COVID-like illness, which reduces the likelihood of bias related to seeking health care or testing, which is an advantage compared to traditional case control and cohort studies [2,27,29]; (3) the vaccination status is usually recorded before performing the test, prior to the knowledge of the result, so that a potential bias of differential misclassification of exposure is avoided [2].

As with other observational studies, this study may have limitations in internal validity that we will try to control in the study design and data analysis.

Vaccinated individuals may have more risk behaviours because they believe they are protected, resulting in underestimation of the vaccine effectiveness [2]. Furthermore, it will not be possible to directly measure vaccine effectiveness against specific variants due to the low proportion of genotyped cases. However, periods in which different variants were dominant will be analysed so that an approximation of vaccine effectiveness against these variants can be achieved, indirectly.

Confounding may occur when the vaccination status is associated with a person’s risk of being exposed to SARS-CoV-2. If, for example, people who choose not to be vaccinated are also those who do not adhere to individual protection measures, this can lead to an overestimation of vaccine effectiveness.

It is also worth noting that participants in research with test-negative designs are limited to those who use health-care services. The National Health Service in Portugal is universal, general and tendentiously free of charge. Although undocumented migrants or refugees may use public healthcare services, they may avoid going to hospitals because of fear or language barriers and will be at higher risk for serious illness in general than other people; this limits the generalizability of the findings to the disadvantaged groups that could not be represented in our study.

## 5. Conclusions

We consider that the findings of this study will have broad implications for understanding the real-world performance of the COVID-19 vaccines at the local level, which may play a key role in promoting adherence to vaccination. Moreover, this study may contribute to inform decisions regarding booster doses, variant-specific vaccine formulations, other pandemic mitigation measures and, ultimately, to the prevention and control of this and future pandemics.

## Data Availability

Not applicable.
